# Making Babbit Metal

**Published:** 1900-04-15

**Authors:** William R. Hall


### MAKING BABBIT METAL.

To make a small quantity of this alloy, a process different from that of the large manufacturers is required, and is in the resources of any dental laboratory. The following is the formula for Dr. Hakell's alloy: Copper 1 oz.; antimony, 2 oz.; and tin 8 oz. The usual directions for melting the metals for making this alloy are to fuse the copper first, the antimony, and the tin last. This is not easily done, as copper requires the heat of a blast furnace for complete fusion. To avoid this trouble, the copper, in the form of nails, is placed in a saturated solution of zinc chlorid; is taken out and dried previous to being used. The tin is divided into two portions; one portion is put in an iron ladle, placed on a coal fire and allowed to come to a dull red heat; if fully dry, the copper nails are thrown in and pushed to the bottom of the ladle; they will soon melt; then follow with the antimony and second portion of the tin; stir all together, and when well mixed remove from the fire.—*William R. Hall, D.D.S., International Dental Journal.*

